# Knockdown of circRNA circ_0087378 Represses the Tumorigenesis and Progression of Esophageal Squamous Cell Carcinoma Through Modulating the miR-140-3p/E2F3 Axis

**DOI:** 10.3389/fonc.2020.607231

**Published:** 2021-02-12

**Authors:** Jing Wang, Qiushuang Wang, Yi Gong, Qiu Hu, Haoliang Zhang, Shaobo Ke, Yongshun Chen

**Affiliations:** ^1^ Department of Clinical Oncology, Renmin Hospital of Wuhan University, The First Clinical College of Wuhan University, Wuhan, China; ^2^ Department of Gastrointestinal Surgery, Renmin Hospital of Wuhan University, The First Clinical College of Wuhan University, Wuhan, China; ^3^ Department of Oncology, Tangshan Workers' Hospital, Tangshan, China

**Keywords:** esophageal squamous cell carcinoma, circ_0087378, miR-140-3p, E2F3, cancer progression

## Abstract

**Background:**

We aimed to investigate the function and underlying mechanisms of circ_0087378 in esophageal squamous cell carcinoma (ESCC).

**Methods:**

We verified higher circ_0087378 expression in ESCC tissues by performing qRT-PCR assays. We further confirmed the oncogenic roles of circ_0087378 in ESCC cells through a series of biological function assays. Then, we used an RNA pull-down assay and luciferase reporter assay to identify miR-140-3p that directly interacts with circ_0087378. Subsequent studies were performed to demonstrate that the circ_0087378/miR-140-3p/E2F3 axis promotes ESCC development.

**Results:**

We demonstrated that upregulated circ_0087378 expression was positively associated with tumor size, histological grade, tumor stage, the presence of metastasis, and worse survival in patients with ESCC. Our results further revealed that knockdown of circ_0087378 suppressed the proliferation, migration, and invasion of ESCC cells and reduced tumor growth *in vivo*. Mechanistically, we showed that circ_0087378 could directly bind to miR-miR-140-3p and relieve the suppression for target E2F3, which accelerated cell proliferation, migration, and invasion. Correlation analysis in ESCC specimens supported the involvement of the circ_0087378/miR-140-3p/E2F3 axis in ESCC progression.

**Conclusions:**

This study demonstrated that circ_0087378 might act as a competing endogenous RNA for miR-140-3p, which could inhibit the tumorigenesis and progression of ESCC through upregulating E2F3 expression.

## Introduction

Esophageal cancer (EC) is one of the most common malignancies worldwide (1, 2). There has been a dramatic rise in the incidence of EC in the developed world over the last 30 years (3). The vast majority of ECs occur as either squamous cell carcinomas (ESCCs) or adenocarcinomas (4, 5). ESCCs account for approximately 90% of EC cases (4, 5). Owing to the lack of inconspicuous early symptoms, most ESCC patients are diagnosed at late-stages (6, 7). Therefore, a better understanding of the molecular mechanisms that drive tumorigenesis and progression in ESCC is critical to develop more effective anti-ESCC treatments.

In recent years, the involvement of small non-coding RNAs, such as circular RNAs (circRNAs) and microRNAs (miRNAs), in ESCC development is increasingly appreciated (8, 9). MiRNAs control gene expression by binding to a complementary sequence in the 3′-untranslated region (3′-UTR) of target mRNAs (10). Until now, miRNAs have been extensively studied in ESCC, where they may be dysregulated and act as biomarkers or therapeutic targets (8, 9). On the other hand, the participation of circRNAs in ESCCs is much less well-established (8, 9). Emerging evidence suggests that some circRNAs function as miRNA sponges to trap miRNA involved in tumorigenesis and metastasis (8, 9). It has been reported that circLPAR3 could target miR-198 to promote ESCC metastasis (11). CircRNA_001275 is a sponge of miR-370-3p to enhance cisplatin resistance in ESCC (12). CircPVT1 could target miR-4663 to promote EC proliferation (6). Circ_0087378 is a newly identified circRNA in breast cancer (13). To date, however, the functions and mechanisms of circ_0087378 in ESCC remain largely unknown.

MiR-140-3p was reported to suppress the malignant properties in colorectal cancer (14), breast cancer (15), and lung cancer (16). However, the role and potential target genes of miR-140-3p in ESCC is still poorly understood. Moreover, E2F3, an important member of the E2F family has been reported to have critical roles in regulating cell cycle, cell differentiation, and apoptosis of human cancer cells (17–19). Accumulating data demonstrated that the LINC00467-miR-200a-E2F3 axis was implicated in the regulation of glioma cell tumorigenesis (20). Additionally, circPRMT5 sponges miR-377 to increase the expression of E2F3 in colorectal cancer (21). However, there are no previous reports that address the function of E2F3 in ESCC. In this study, we have proved that circ_0087378, as a newly discovered circRNA in ESCC, could sponge miR-140-3p to upregulate the expression of E2F3 and eventually serve as a tumor promotor in ESCC.

## Materials and Methods

### Patient Samples

This study has been approved by the Medical Ethics Committee of Wuhan University People’s Hospital and conformed to the declaration of Helsinki. A cohort of ESCC tissues and matched normal tissues was derived from 50 patients with ESCC. These patients did not receive radiotherapy or chemotherapy before surgery. All patients provided their informed consent in writing before they participated in this study. Tumor tissues and adjacent non-cancerous tissues were stored at a −80°C freezer.

### Cell Lines and Culture Conditions

The ESCC cell lines (KYSE41, EC109, EC9706, KYSE150, and KYSE30) and the immortalized human esophageal epithelial cell line SHEE were obtained from the Cell Bank of Type Culture Collection of Chinese Academy of Sciences (Shanghai, China). Cells were cultured in RPMI-1640 medium (Solarbio, China) supplemented with 10% fetal bovine serum, 100 mg/ml streptomycin, and penicillin at 37°C in 5% CO2.

### Lentiviral Vectors and Transfection

Short hairpin RNAs (shRNA) targeting circ_0087378 (circ_0087378#1 and sh-circ_0087378#2) and the negative control shRNA (shNC) were obtained from GenePharma (Shanghai, China). All shRNA sequences were listed in **Table 1**. HEK-293T cells were co-transfected with the shRNA plasmids, psPAX2, and pMD2.G. The supernatant containing the lentivirus particles was used to infect EC109 or KYSE150 cells for 48 h, and transduced cells were selected by addition of puromycin (2 μg/ml; Sigma-Aldrich, USA). The negative control miRNA mimic (miR-NC) and miR-140-3p mimic (miR-140-3p), negative control miRNA inhibitor (anti-miR-NC) and miR-140-3p inhibitor (anti-miR-140-3p) were purchased from GenePharma. Full-length cDNA of E2F3 was inserted into the pcDNA3.1 vector to generate the E2F3 overexpression plasmid (Honorgene, Changsha, China). MiRNA mimic, miRNA inhibitor, and plasmids were transfected using Lipofectamine 3000 (Invitrogen, Carlsbad, CA, USA) according to the manufacturer’s instructions.

**Table 1:** shRNA sequence for human circ_0087378.

Name	Primer sequence (5’-3’)
shNC	UUCUCCGAACGUGUCACGUTT
sh-circ_0087378#1	GGCATGAAAGGTGCAAACCCA
sh-circ_0087378#2	GCATGAAAGGTGCAAACCCAA

### Quantitative Real-Time PCR (qRT-PCR) Assay

TRIzol reagent was purchased from Invitrogen (Carlsbad, CA, USA) and employed to isolate total RNAs. Reverse Transcriptase Kit was obtained from Takara (Beijing, China) and employed to reverse transcribed the circRNA and mRNA. Total RNA was also reverse-transcribed by a MicroRNA Reverse Transcription Kit (Applied Biosystems, Foster City, CA, USA). The qRT-PCR assays were performed as previously reported (12). GAPDH and U6 were used as internal controls for circRNAs and miRNAs, respectively. All primer sequences were listed in **Table 2**.

**Table 2:** Primers used for qRT-PCR assay.

Target	Primer sequence (5’-3’)
circ_0087378-hF	TCTCGGTCTATGCTGTGGTG
circ_0087378-hR	CATTCGCTGCAGTTCCATAA
miR-140-3p-hF	CAGTGCTGTACCACAGGGTAGA
miR-140-3p -hR	TATCCTTGTTCACGACTCCTTCAC
E2F3-hF	TATCCCTAAACCCGCTTCC
E2F3-hR	TTCACAAACGGTCCTTCTA
GAPDH -hF	TGAACGGGAAGCTCACTGG
GAPDH -hR	TCCACCACCCTGTTGCTGTA
U6-hF	CTCGCTTCGGCAGCACA
U6-hR	AACGCTTCACGAATTTGCGT

### RNase R Treatment

RNase R (Epicentre Company) was used to eliminate the linear RNA. The expression of GAPDH and circ_0087378 was assessed using qRT-PCR assays.

### Cell Proliferation Assay

Cell proliferation test was performed according to CCK-8 manuscript on day 0, day 1, day 2, and day 3 after transfection.

### Colony Formation Assay

EC109 or KYSE150 cells (1 × 10^3^/well) were seeded into a six-well plate. After 14 days, cells were fixed with 4% paraformaldehyde and then stained by 0.1% crystal violet. After that, the number of clones was counted and analyzed.

### Cell Migration and Invasion Assays

Transwell cell migration and invasion assays were carried out as previously described (22). In brief, EC109 or KYSE150 cells (1 × 10^5^) were added to the upper chambers of transwell plates. The lower chambers were filled with 750 μl of RPMI-1640 medium containing 10% FBS. After an incubation period of 24 h, the migrated or invaded cells were stained by 0.1% crystal violet for 30 min. The number of cells was calculated from 10 random fields in each chamber.

### Flow Cytometry Analysis

EC109 or KYSE150 cells (1× 10^5^/well) were harvested and stained using an Annexin V-FITC apoptosis detection kit (Solarbio). The cells were then analyzed using a FACSCalibur flow cytometer (BD Biosciences, USA).

### ESCC Xenograft Mouse Model

Fifty 6–8 weeks old BALB/c nude mice (20 g) were purchased from Shanghai Laboratory Animals Center (China) and employed to establish the mouse models as previously reported (23). In brief, 2 × 10^5^/20 µl of ESCC cells were subcutaneously injected into the nude mice. The tumor volume of each mouse was measured every 7 days. All procedures and animal experiments were approved by the Animal ethics committee of Wuhan University.

### Dual-Luciferase Reporter Assay, RNA Pull-Down Assay, and RIP Assay

The Dual-luciferase reporter gene assay, RNA pull-down and RIP assays were performed as previously described (24, 25). Briefly, the luciferase reporter plasmids containing circ_0087378-wild type (WT), circ_0087378-muatnt (MUT), E2F3-WT or E2F3-MUT were produced from the pmirGLO vectors (Promega, Madison, WI, USA). Primer sequences were as follows: circ_0087378-WT (Forward primer: 5′-GGAATTCCTGTGGTGGTGATTGCGTCTGTGGT-3′; Reverse primer: 5′-CCAAGCTTGG CTTTCATGCCAAACTTGGAGTG-3′), circ_0087378-MUT (Forward primer: 5′-GGAATTCCTGTGATAATGATTGCGTTTATGGT-3′; Reverse primer: 5′-CCAAGCTTGG CTTTCATGCCAAACTTGGAGTG-3′), E2F3 3′-UTR-WT (Forward primer: 5′-GGAATTCCGGTACAAAATGTCGGTGTGGTC-3′; Reverse primer: 5′-CCAAGCTTGGGCCAGGTGAGCTCAGTCTTT-3′), and E2F3 3′-UTR-MUT (Forward primer: 5′-GGAATTCCGGTACAAAATGTCGGTCTAGCC-3′; Reverse primer: 5′-CCAAGCTTGGGCCAGGTGAGCTCAGTCTTT-3′). EC109 or KYSE150 cells were co-transfected with the above reporter plasmids, with miR-140-3p mimic or miR-NC, respectively. The luciferase signal was measured using a Dual-Luciferase Reporter Assay System (Promega). EC109 or KYSE150 cells were transfected with the biotin-labeled circ_0087378 (Bio- circ_0087378-wt) or the respective control. Cells were digested after 48 h, and streptavidin magnetic beads were used to incubate the lysates. The expression of miR-140-3p was measured by qRT-PCR assay. RIP was performed using an EZ-Magna RIP Kit (Millipore, Billerica, MA, USA). EC109 or KYSE150 cells were lysed using the RIP lysis buffer (Sigma-Aldrich, USA). Magnetic beads coupled with anti-immunoglobulin G (IgG) or anti-Argonaute 2 (AGO2) were employed to incubate cell lysates. The expression of circ_0087378 and miR-140-3p in immunoprecipitated RNAs were assessed by qRT-PCR assay.

### Western Blotting Analysis

EC109 and KYSE150 cells were lysed by RIPA lysis buffer (Beyotime, Shanghai, China) The protein was isolated and transferred onto PVDF membranes as previously described (26). After blocking 5% milk, the membranes were incubated with primary antibodies (**Table 3**). Then the membranes were incubated with the secondary antibodies. Immunoblot signals were visualized using the EasyBlot ECL kit (Sangon Biotech, China). Detected bands were quantified using ImageJ software (Rawak Software, Inc. Germany).

**Table 3:** List of antibodies used in western blotting analysis.

Antibody Name	Company	Dilution
Anti-E2F3 (ab152126)	Abcam	1:2,000
Anti-GAPDH (ab9484)	Abcam	1:2,000

### Statistical Analysis

Statistical analysis was performed using SPSS 21.0 software (IBM, USA). All the experiments were repeated three times. All values were presented as means ± standard deviation (SD). The difference was calculated by the Student’s *t*-tests, the *χ* ^2^-tests and one-way ANOVA analysis. Kaplan-Meier analysis was used to evaluate overall survival, and a Spearman correlation coefficient was used to analyze correlations. A *P*-value of <0.05 was considered statistically significant.

## Results

### Circ_0087378 Is Upregulated in ESCC Tissues and Higher circ_0087378 Was Correlated With Poorer Outcomes in ESCC Patients

**Figure 1:**
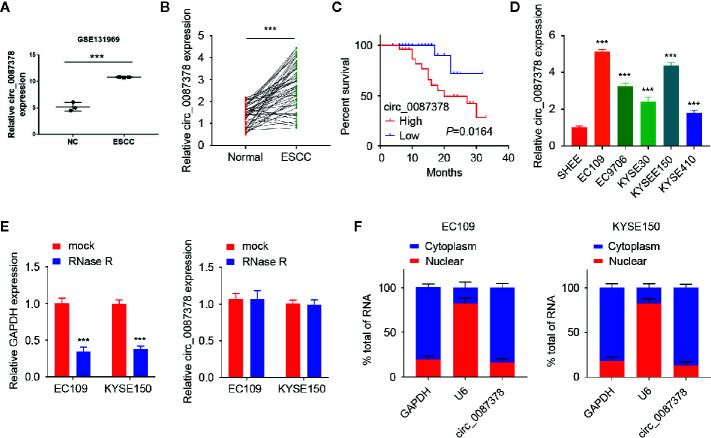
Circ_0087378 is upregulated in ESCC tissues and higher circ_0087378 was correlated with poorer outcomes in ESCC patients. **(A)** Circ_0087378 expression in ESCC tissues and normal tissues (GSE131969). **(B)** Circ_0087378 expression in ESCC tissues and adjacent normal tissues (n = 50 specimens). **(C)** Kaplan-Meier analysis of overall survival in ESCC patients with high *versus* low circ_0087378 levels. **(D)** The expression of circ_0087378 in ESCC cell lines and the immortalized human esophageal epithelial cell line SHEE were measured using the qRT-PCR assay. **(E)** The levels of circ_0087378 and linear GAPDH were detected by using the qRT-PCR assay in EC109 and KYSE150 cells treated with RNase R. **(F)** Quantitation of nuclear and cytoplasmic fractions showed that circ_0087378 was mainly located in the cytoplasm. ***P < 0.001.

In order to seek for circRNAs aberrantly expressed in ESCC, we analyzed the GEO dataset (GSE131969) containing three ESCC tissues and three normal tissues and found that circ_0087378 is highly expressed in ESCC tissues relative to adjacent normal tissues (**Figure 1A**). By using the qRT-PCR assays, we verified the upregulation of circ_0087378 expression in ESCC tissues compared with adjacent normal tissues (**Figure 1B**). Consistent with these results, we found that higher circ_0087378 expression was positively correlated with tumor size, histological grade, tumor stage, and the presence of metastasis in patients with ESCC (**Table 4**). Based on the median expression value of circ_0087378 in 50 ESCC cancer tissues, patients were divided into circ_0087378-low expression group and circ_0087378-high expression group. Kaplan-Meier survival analysis showed that patients with higher circ_0087378 levels displayed lower overall survival rates (**Figure 1C**).

**Table 4:** Correlations of circ_0087378, miR-140-3p, and E2F3 expression with clinicopathologic features of esophageal squamous cell carcinoma.

Factor	circ_0087378 expression			*P* value	miR-140-3p expression		*P* value	E2F3 expression		*P* value
		Low (n = 25)	High (n = 25)		Low (n = 25)	High (n = 25)		Low (n = 25)	High (n = 25)	
Age				0.395			0.156			0.284
≤65		15	12		16	11		13	14	
>65		10	13		9	14		12	11	
Gender				0.569			>0.99			0.087
Male		13	15		14	14		11	17	
Female		12	10		11	11		14	8	
Tumor size				0.024			0.258			0.005
≤2cm		16	8		10	14		17	7	
>2cm		9	17		15	11		8	18	
Tumor differentiation				0.004			0.001			0.023
Well		16	6		5	17		15	7	
Poor		9	19		20	8		10	18	
TNM stage				0.048			0.011			0.019
I/II		16	9		6	19		18	7	
III/IV		9	16		19	6		7	18	
Lymph node metastasis				0.012			0.002			0.529
Negative		22	14		16	20		19	17	
Positive		3	11		9	5		6	8	
Distant metastasis				<0.001			0.005			0.031
Yes		2	13		18	8		4	11	
No		23	12		7	17		21	14	

Then, we assessed the expression of circ_0087378 in various ESCC cell lines. Circ_0087378 expression was significantly higher in human ESCC cell lines than that in human esophageal epithelial cell line SHEE (**Figure 1D**). Compared to linear GAPDH mRNA, circ_0087378 was found to be resistant to RNase R (**Figure 1E**). We further analyzed the subcellular location of circ_0087378. Our results suggested that circ_0087378 was mainly located in the cytoplasm (**Figure 1F**). Taken together, our data supported that circ_0087378 was highly expressed in ESCC, and higher circ_0087378 was correlated with poorer outcomes in ESCC patients.

### Knockdown of circ_0087378 Suppresses ESCC Progression *In vitro* and *In Vivo*

**Figure 2:**
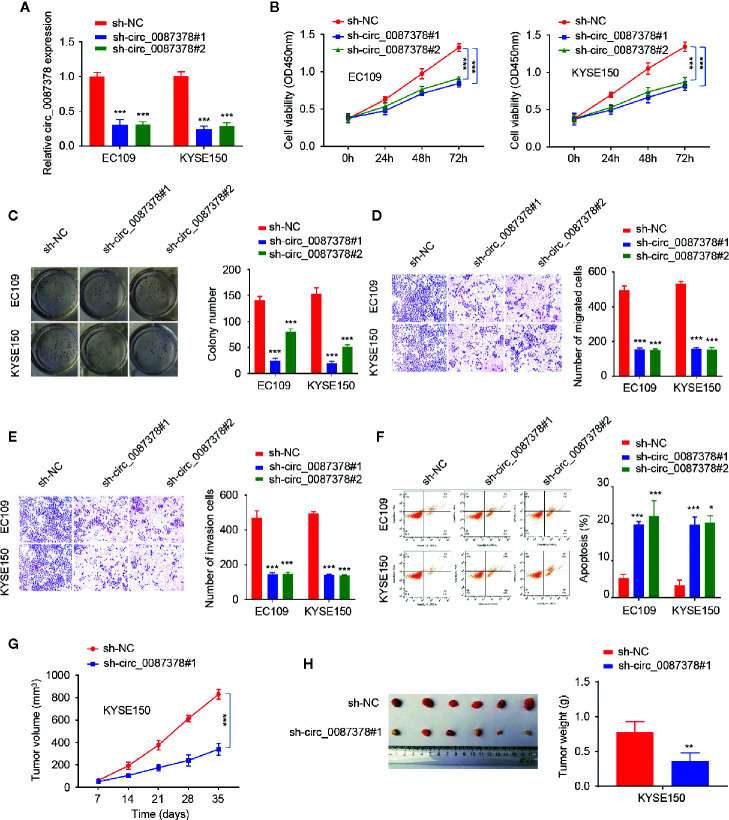
Knockdown of circ_0087378 Suppresses ESCC Progression *in vitro* and *in vivo.* ***(*A*)*** Expression of circ_0087378 in EC109 and KYSE150 cells transfected with indicated siRNAs. **(B, C)** CCK-8 assays **(B)** and clone colony formation assays **(C)** in ESCC cells after knockdown of circ_0087378. **(D, E)** Cell migration **(D)** and cell invasion **(E)** assays in ESCC cells after circ_0087378 knockdown. **(F)** Flow cytometry analysis was used to detect ESCC cell apoptosis after knockdown of circ_0087378. **(G, H)** KYSE150 cells transfected with (or without) sh-circ0087378#1 were injected into nude mice. **(G)** Tumor volumes of xenograft tumors were measured. **(H)** Representative images of tumors and tumor weights of xenograft tumors. *P < 0.05, **P < 0.01, ***P < 0.001.

Of the ESCC cell lines, circ_0087378 expression was highest in the EC109 and KYSE150 cell lines (**Figure 1D**), which were selected for subsequent circ_0087378-knockdown experiments. Short hairpin RNAs (shRNA) targeting the back-spliced section of circ_0087378 (sh-circ_0087378#1 and sh-circ_0087378#2) were generated to effectively knockdown circ_0087378 expression in EC109 and KYSE150 cells (**Figure 2A**).

We next evaluated the biological role of circ_0087378 in ESCC. The results demonstrated that depletion of circ_0087378 with shRNAs could significantly inhibit the proliferation, migration, and invasion of ESCC cell lines (**Figures 2B–E**). Flow cytometry analysis showed that knockdown of circ_0087378 in ESCC cells significantly induced cell apoptosis (**Figure 2F**). Also, we established a nude mice model of ESCC by subcutaneous inoculation of ESCC cells transfected sh-circ_0087378#1 or mock cells. We found that the downregulation of circ_0087378 resulted in a significantly lower tumor size and weight compared with controls (**Figures 2G, H**). These data suggested that silencing of circ_0087378 significantly inhibited the progression of xenograft tumors possibly through inducing apoptosis *in vivo.*

### Circ_0087378 Sequesters MiR-140-3p in ESCC Cells

**Figure 3:**
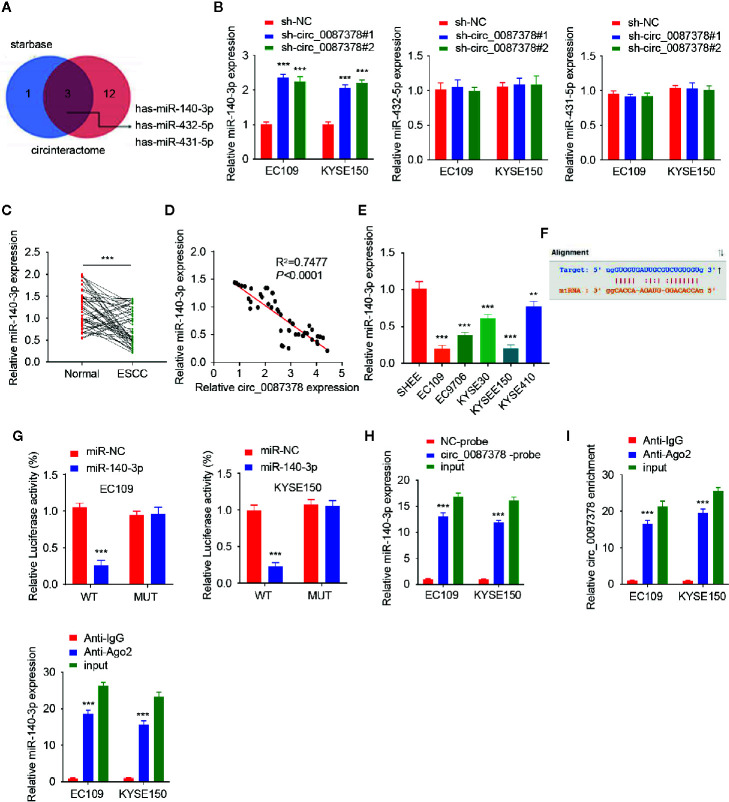
Circ_0087378 Sequesters MiR-140-3p in ESCC cells. **(A)** We used the StarBase and CircInteractome databases to select the top three common miRNAs based on their conjugation scores. **(B)** The expression of three miRNAs in ESCC cells after knockdown of circ_0087378. **(C)** The expression of miR-140-3p in ESCC tissues and adjacent normal tissues. **(D)** The negative correlation between miR-140-3p and circ_0087378 in ESCC tissues, as determined by the qRT-PCR assay. **(E)** The expression of miR-140-3p in ESCC cell lines and SHEE cells were measured using qRT-PCR assay. **(F)** Computational prediction of the interaction between miR-140-3p and circ_0087378 sequence. **(G)** Luciferase reporter assays in ESCC cells transfected with circ_0087378-WT or circ_0087378-MUT, along with miR-140-3p mimic or control mimic. **(H, I)** RNA pull-down assays **(H)** and RIP-qRT-PCR assays **(I)** were used to confirm the interaction between circ_0087378 and miR-140-3p in ESCC cells. ***P < 0.001.

Given that circRNAs have been reported to act as miRNA sponges (8, 9), we wonder whether circ_0087378 can bind to certain miRNAs in the progression of ESCC. We predicted the miRNAs that might target circ_0087378 by analyzing the StarBase and CircInteractome databases. We then selected the top three miRNAs (including miR-140-3p, miR-431-5p, and miR-432-5p) based on conjugation scores (**Figure 3A**). Our qRT-PCR assays determined that knocking down circ_0087378 in ESCC cells significantly upregulated the expression of miR-140-3p (but not the remaining two miRNAs) (**Figure 3B**). Subsequently, we confirmed the downregulation of miR-140-3p in ESCC tissues compared with adjacent normal tissues (**Figure 3C**). Furthermore, we detected the negative correlation between the expression of circ_0087378 and miR-140-3p in ESCC tissues by performing the qRT-PCR analysis (**Figure 3D**). As expected, the levels of miR-140-3p were downregulated in ESCC cells compared with SHEE cells (**Figure 3E**).

Next, we utilized a luciferase reporter assay to verify the binding of miR-140-3p to circ_0087378 in ESCC cells (**Figure 3F**). Overexpression of miR-140-3p inhibited the luciferase activity of circ_0087378-WT, but did not impact the luciferase activity of the circ_0087378-MUT (**Figure 3G**). We conducted an RNA pull-down assay using a biotin-tagged circ_0087378 probe and found that miR-140-43p was purified with the biotin-labeled circ_0087378 probe compared with the negative control (**Figure 3H**). After that, we performed the RIP assay to pull down circ_0087378 and miR-140-3p in ESCC cells using anti-Ago2 antibodies or control IgG, following by qRT-PCR analysis. The results showed that circ_0087378 and miR-140-3p were significantly enriched in the Ago2 IP fraction in comparison to the IgG control fractions (**Figure 3I**). Collectively, these results supported a direct interaction between circ_0087378 and miR-140-3p in ESCC cells.

### Circ_0087378 Promotes the Proliferation and Invasiveness of ESCC Cells by Sponging miR-140-3p

**Figure 4:**
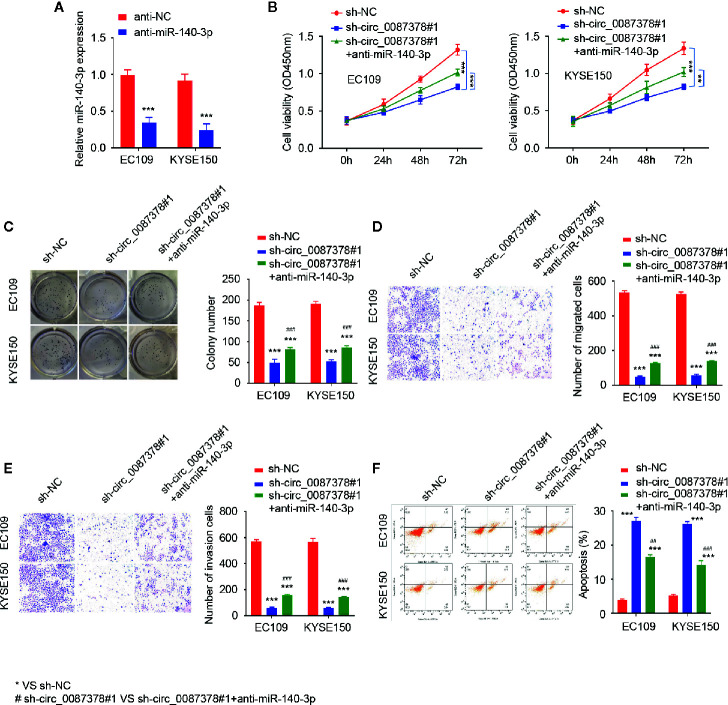
Circ_0087378 promotes the proliferation and invasiveness of ESCC cells by sponging miR-140-3p. **(A)** The expression of miR-140-3p in ESCC cells transfected with anti-miR-140-3p inhibitor or the control inhibitor. **(B–F)** CCK-8 assays **(B)**, clone colony formation **(C)**, cell migration **(D)**, cell invasion **(E)** assays and Flow cytometry analysis **(F)** in ESCC cells transfected with (or without) sh-circ_0087378#1, along with (or without) anti-miR-140-3p inhibitor. *P < 0.05, **P < 0.01, ***P < 0.001; ^#^ *P* < 0.05, ^##^ *P* < 0.01, ^###^ *P* < 0.001.

We questioned whether circ_0087378 exerts its tumor-promoting effects *via* sponging miR-140-3p by performing rescue experiments. The transfection with anti-miR-140-3p inhibitor significantly decreased the levels of miR-140-3p in ESCC cells (**Figure 4A**). Subsequent cell functional assays demonstrated that the inhibition of miR-140-3p restored the proliferative and invasive properties of ESCC cells that were produced by circ_0087378 knockdown (**Figures 4B–F**). The flow cytometry analysis indicated that the knockdown of circ_0087378 increased cell apoptosis, while co-transfection with anti-miR-1403p significantly reduced cell apoptosis compared with the control (**Figure 4F**). The downregulation of miR-140-3p was more likely to occur in patients with high tumor grade, late-stage, and a high risk of lymph node metastasis (**Table 4**). These results suggested that circ_0087378 promotes the proliferation and invasiveness of ESCC cells by sponging miR-140-3p.

### MiR-140-3p Inhibits the Proliferation and Invasion of ESCC Cells by Targeting E2F3

**Figure 5:**
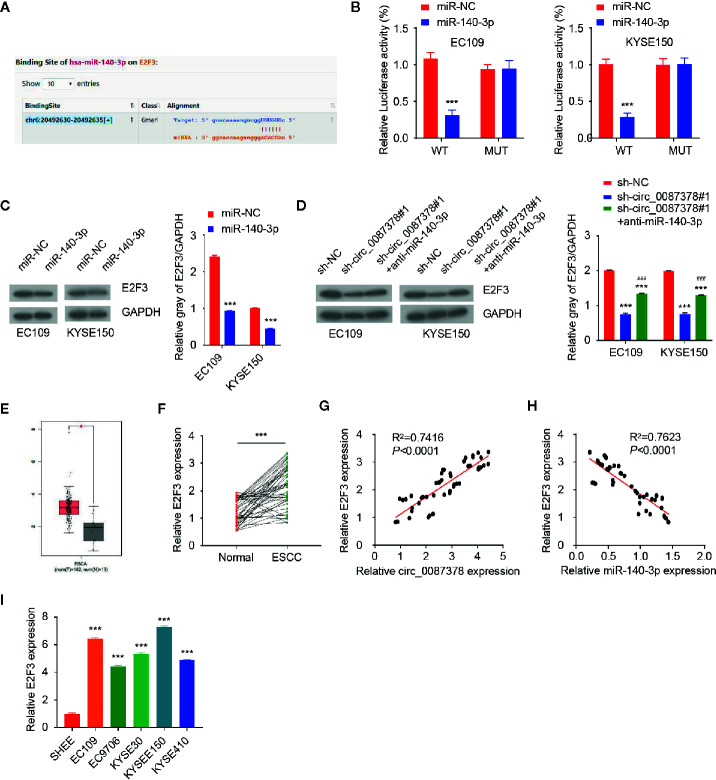
MiR-140-3p directly targets E2F3 in ESCC cells. **(A)** Computational prediction of interaction between miR-140-3p with the E2F3 3′-UTR sequence. **(B)** Luciferase reporter assays in ESCC cells transfected with E2F3-WT or E2F3-MUT, along with miR-140-3p mimic or control mimic. **(C)** Western blotting analysis of E2F3 expression in ESCC cells transfected with (or without) miR-140-3p mimic. **(D)** Western blotting analysis of E2F3 levels in ESCC cells co-transfected with (or without) miR-140-3p, along with (or without) the E2F3 expression vector. **(E)** The expression of E2F3 in TCGA ESCC tissues and normal tissues. **(F)** qRT-PCR analysis of E2F3 expression in ESCC tissues and adjacent normal tissues. **(G)** The positive correlation between circ_0087378 and E2F3 in ESCC tissues was determined by qRT-PCR assays. **(H)** The negative correlation between miR-140-3p and E2F3 in ESCC tissues was determined by qRT-PCR assay. **(I)** E2F3 expression in ESCC cell lines and SHEE cells was measured using qRT-PCR assays. ***P < 0.001; ^###^ *P* < 0.001.

Since these data indicated that circ_0087378 enhances the aggressive features of ESCC cells *via* miR-140-3p sequestration, we sought to determine whether circ_0087378 could increase the expression of miR-140-3p target genes. We queried the StarBase database to identify miR-140-3p target genes, and discovered a miR-140-3p binding site on the 3′-UTR of *E2F3* mRNA (**Figure 5A**). To validate whether E2F3 is a miR-140-3p target gene, ESCC cells were co-transfected with E2F3 3′-UTR-WT or E2F3 3′-UTR-MUT, along with (or without) miR-140-3p mimic. As a result, miR-140-3p mimic significantly decreased the luciferase activities of E2F3 3′-UTR-WT (**Figure 5B**). However, the transfection with miR-140-3p mimic did not significantly impact the luciferase signal of E2F3 3′-UTR-MUT (**Figure 5B**). Moreover, ESCC cells transfected with miR-140-3p mimic expressed lower E2F3 protein levels (**Figure 5C**). Our further studies showed that silencing of circ_0087378 could lead to an apparent downregulation of E2F3 (**Figure 5D**). However, these changes were largely prevented after the transfection with anti-miR-140-3p inhibitor (**Figure 5D**). These results revealed that circ_0087378 promoted the expression of E2F3 in ESCC cells by reducing the inhibitory effects of miR-140-3p.

**Figure 6:**
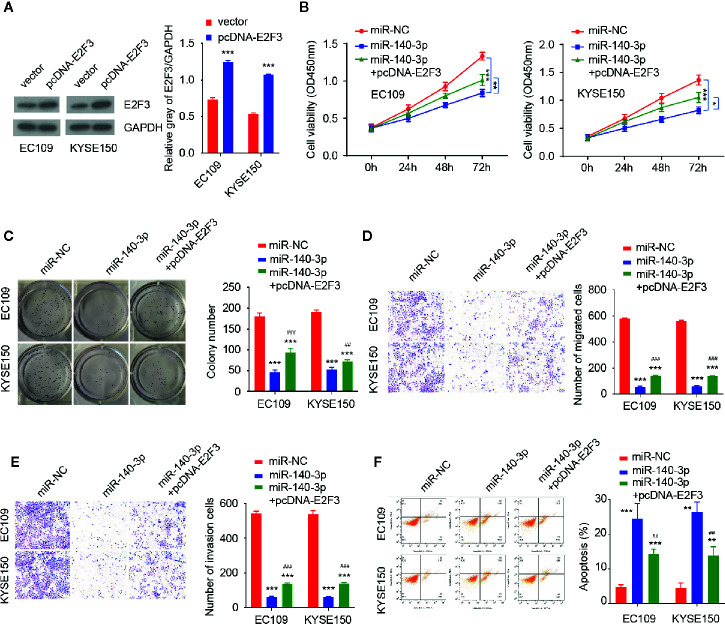
E2F3 could reverse the inhibitory effects of miR-140-3p in ESCC cells **(A)** Western blotting analysis of E2F3 levels in ESCC cells transfected with (or without) the E2F3 expression vector. **(B–F)** CCK-8 assays **(B)** and clone colony formation assays (**C**), Cell migration (**D**), cell invasion **(E)** assays and Flow cytometry analysis **(F)** in ESCC cells co-transfected with (or without) miR-140-3p mimic, along with (or without) the E2F3 expression vector. *P < 0.05, **P < 0.01, ***P < 0.001; ^#^ *P* < 0.05, ^##^ *P* < 0.01, ^###^ *P* < 0.001.

We compared the expression of E2F3 in ESCC tissues and normal tissues by using the TCGA ESCC dataset. E2F3 levels were significantly higher in ESCC tissues *versus* normal tissues (**Figure 5E**). Our qRT-PCR assays revealed an upregulation of E2F3 in ESCC tumor specimens compared with adjacent normal tissues (**Figure 5F**). In agreement with these findings, the levels of circ_0087378 were positively correlated to the expression of E2F3 expression in ESCC tissue (n = 50) (**Figure 5G**). Meanwhile, highly expressed E2F3 was negatively correlated with lower miR-140-3p expression (**Figure 5H**). Compared with SHEE cells, the expression of E2F3 was clearly upregulated in ESCC cell lines (**Figure 5I**). Consistent with these results, we found that higher E2F3 expression was positively correlated with large tumor size, high tumor grade, late tumor stage, and the presence of metastasis in patients with ESCC (**Table 4**). Furthermore, overexpression of miR-140-3p led to decreased cell growth, cell migration and invasion, as well as increased cell apoptosis (**Figures 6A–F**). However, these effects could be significantly abolished by ectopic overexpression of E2F3 (**Figures 6A–F**). These data support the notion that the circ_0087378/miR-140-3p/E2F3 axis is essential for ESCC growth and progression.

## Discussion

Recent studies have shown that miRNAs and circRNAs participate in the regulation of ESCC initiation and progression (8, 9, 27). For example, circ_0000337 could sponge miR-670-5p to regulate the migration and invasion of ESCC cells (28). In addition, circ_0000337 promotes ESCC metastasis by augmenting beta-catenin signaling (29). To date, there were few studies on circ_0087378 function in ESCC. In this study, we found that circ_0087378 was highly expressed in ESCC tumor tissues relative to adjacent normal tissues. Our *in vitro* and *in vivo* results demonstrated that circ_0087378 performed pro-oncogenic functions in ESCC. Our results suggest, for the first time, the importance of circ_0000337 as a key regulator in human ESCC progression.

Several previous studies have shown that miR-140-3p is a critical miRNA that acts as a tumor suppressor in various cancers (30–32). Furthermore, miR-140-3p was shown to be down-regulated in ESCC tissues (21). MiR-140-3p could interact with NRIP1 to inhibit the tumorigenesis of ESCC (24). Consistent with these results, we demonstrated that circ_0000337 promoted the proliferation and invasion of ESCC cells *via* sponging miR-140-3p.

As an important member of the E2F family, E2F3 affects tumorigenesis and progression in lung cancer (33) and osteosarcoma (34). Furthermore, E2F3 was up-regulated in ESCC tissues (35). Here, our results verified that miR-140-3p directly binds to E2F3 and inhibits its expression in ESCC cells. E2F3 enhances the malignant properties of ESCC cells, and overexpression of E2F3 could reverse the tumor-suppressing functions of miR-140-3p. Moreover, the expression of E2F3 was significantly positively correlated with the levels of circ_0087378, while E2F3 expression was negatively correlated with the levels of miR-140-3p in ESCC tumor tissues. Taken together, our findings demonstrated that circ_0087378 promoted the tumorigenesis and progression of ESCC *via* regulating the miR-140-3p/E2F3 axis.

There were certain limitations in our study. We only tested the effects of circ_0087378 knockdown, but not the influence of circ_0087378 overexpression on ESCC cells. In addition, our results showed that circ_0087378 inhibits the function of miR-140-3p, while inhibiting miR-140-3p expression failed to completely eliminate the effects of circ_0087378 knockdown on ESCC cells. This might be involved in the complexity of the circ_0087378-miRNA regulatory networks. A previous study has shown that one circRNA can simultaneously target several miRNAs (36), implying that circ_0087378 might target multiple miRNAs in ESCC cells. Thus, reducing the levels of miR-1403-p may not be enough to abolish the effects of circ_0087378 knockdown on ESCC cells. Also, one miRNA was able to bind several targets simultaneously (37). This might account for why overexpressing E2F3 expression did not completely reverse the effects of miR-140-3p on ESCC cells.

In conclusion, our results provide new evidence showing that circ_0087378 is a novel oncogenic circRNA that exerts its tumor-promoting activities in ESCC through mediating the miR-140-3p/E2F3 axis. This study may offer a potential therapeutic target, circ_0087378, to broaden the treatment options for human ESCC.

## Data Availability Statement

Publicly available datasets were analyzed in this study. These data can be found here: the NCBI Gene Expression Omnibus (GSE131969).

## Ethics Statement

The studies involving human participants were reviewed and approved by Renmin Hospital of Wuhan University. The patients/participants provided their written informed consent to participate in this study. The animal study was reviewed and approved by the animal ethics committee of Wuhan University.

## Author Contributions

YC designed the experiments. SK applied the funding. HZ analyzed the public database, designed the study and edited the paper. JW, QW, and YG performed the experiments, HQ analyzed the data. SK and YC wrote the paper. All authors contributed to the article and approved the submitted version.

## Funding

This work was supported by the National Natural Science Foundation of China (No. U1604175), National Key R&D Program of China (2018YFC1311300), Independent research project of Wuhan University (2042019kf0103), and the Beijing Kangmeng Charity Foundation (2020HX0026).

## Conflict of Interest

The authors declare that the research was conducted in the absence of any commercial or financial relationships that could be construed as a potential conflict of interest.
